# Convulsions Cured by Lancing the Gums

**Published:** 1852-10

**Authors:** V. M. Swazye


### ARTICLE XII.

*Convulsions Cured by Lancing the Gums.* By V. M. SWAZYE, D. D. S.

MESSRS. EDITORS:—As there may be members of the dental profession who do not appreciate the benefit often to be derived from lancing the gums in difficult dentition, I send you the following interesting case:

In July, 1847, while on a visit to some relations in Canada, I was requested by Mr. S., who had called at my uncle's, with whom I was staying, to visit a child of his, which had been sick for several weeks, and the symptoms were becoming more and more aggravated every day. During the last three or four days, it had had several convulsions. The illness was supposed to result from teething. When I saw the child, the parents had nearly despaired of its recovery.

On examining the mouth of the child, I become convinced, from the inflamed and swollen condition of its gums, that the constitutional disturbance was caused by irritation produced by the advancing teeth. The child had a convulsion soon after I saw it; and as an old woman, a sort of doctress of the neighborhood, had predicted the day before, that it would die the next day, between ten and twelve o'clock, saying, that mortification of its bowels had taken place, the afflicted mother supposed the infant was in the agonies of death. But the old woman seemed rather happy than otherwise, that her prediction was about to be fulfilled.

Believing that lancing the gums freely down to the advancing teeth, was the only remedy that promised any relief, I took the child from the cradle, and with the consent of the mother, performed this operation. It was relieved, almost instantly, and immediately, on being taken by the mother, it took the breast, and was soon lost in a quiet sleep. Its recovery from this time was uninterrupted, and rapid.

The above case is similar to one related by Professor Harris, to the students of the Baltimore College of Dental Surgery, while the writer was attending lectures in that institution, during the session of 1845-6. I would ask, in conclusion, what causes the death of ten thousand children, annually, in the city of New York, alone? Has not morbid dentition something to do with this fearful mortality? In cases like the foregoing, medicine may afford temporary, but will seldom give permanent, relief.

